# Supplementary material for: Neonatal Mesenchymal Stem Cell Treatment Improves Myelination Impaired by Global Perinatal Asphyxia in Rats
Source: Int J Mol Sci. 2021 Mar 23;22(6):3275. doi: 10.3390/ijms22063275 (PMC8004671; doi:10.3390/ijms22063275)
Supplement: Supplementary file 1 [file ijms-22-03275-s001.pdf]

TapiaBustosetal2021SUPPLMATERIAL

"Neonatal mesenchymal stem cell treatment improves myelination impaired by global perinatal asphyxia in rats"

Tapia-Bustos A[1,2]; Lespay-Rebolledo C[1]; Vío V[1]; Perez-Lobos R[1], Casanova E[1]; Ezquer F[3]; Herrera-Marschitz M[1*], Morales P[1,4*]

[1]Programme of Molecular & Clinical Pharmacology, ICBM; [2]School of Pharmacy, Faculty of Medicine, Universidad Andres Bello, Santiago, Chile; [3]*Centro de Medicina Regenerativa, Facultad de Medicina Clínica Alemana-Universidad del Desarrollo*, Las Condes; [4]Dept. Neuroscience, Medical Faculty, University of Chile, Independencia.

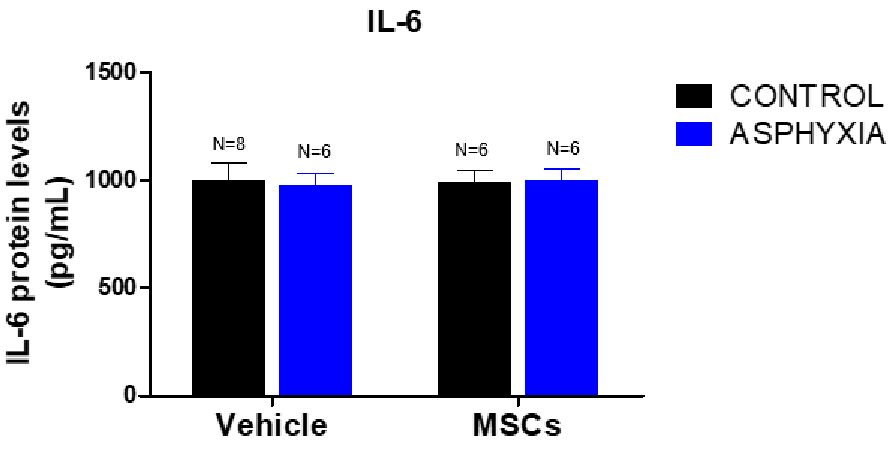

Supplementary Figure 1. Effect of MSCs treatment on changes of IL-6 protein levels induced by perinatal asphyxia (PA), measured at P7 in telencephalon of CS and AS rat neonates: MSCs treatment. Effect of PA on IL-6 protein levels (pg/mL) was determined by ELISA analysis. Data are shown as means ± S.E.M., for independent experiments (N=6-8). In telencephalon, unbalanced two-way ANOVA indicated no significant effect of PA on IL-6 protein levels. Benjamini-Hochberg was used as a post hoc test.

A.

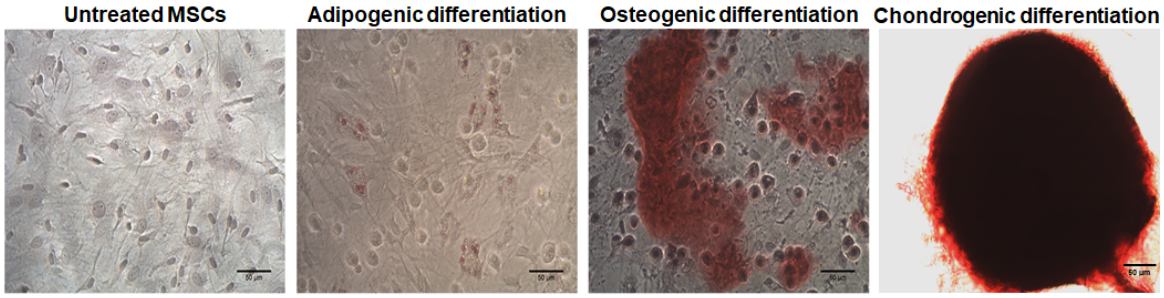

B.

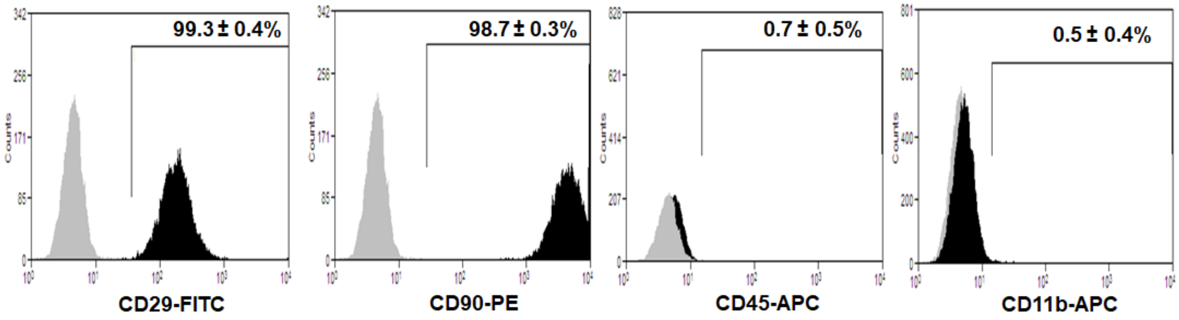

Supplementary Figure 2: Rat adipose-derived MSCs display mesenchymal stem cell characteristics. (A) Representative microphotographs of plastic-adherent cells isolated from rat adipose tissue differentiated into adipogenic, osteogenic or chondrogenic lineages. (B) Immuno-phenotypification of adherent cells according to the expression of putative murine MSCs markers (CD29 and CD90), and the non-expression of markers characteristics of other cell types (CD45 and CD11b). Black histograms represent cells labeled with specific antibodies; grey histograms represent cells stained with isotype control antibodies. FITC: fluorescein isothiocyanate, PE: phycoerythrin, APC: allophycocyanin.

Supplementary Table 1. Specific primers for RT-qPCR amplification.

**Genes**	**Primer Forwards**	**Primer Reverse**	**Amplicon size**
MBP	AGTCGCAGAGGACCCAAGAT	GACAGGCCTCTCCCCTTTC	103 bp
Olig-1	GCCCAGGCCACGAGTACAAA	TCCACTCCGAAACCCAACGA	121 bp
Olig-2	GAAATGGAATAATCCCGAACTACT	CCCCTCCCAAATAACTCAAAC	232 bp
Cox-2	GTTTGGAACAGTCGCTCGTCATC	TGTATGCTACCATCTGGCTTCGG	94 bp
TNF-α	AAATGGGCTCCCTCTCATCAGTTC	TCTGCTTGGTGGTTTGCTACGAC	111 bp
IL-1β	CACCTCTCAAGCAGAGCACAG	GGGTTCCATGGTGAAGTCAAC	79 bp
IL-6	TCCTACCCCAACTTCCAATGCTC	TTGGATGGTCTTGGTCCTTAGCC	79 bp
β-actin	AAGTCCCTCACCCTCCCAAAAG	AAGCAATGCTGTCACCTTCCC	97 bp
